# Early prediction of pathological complete response to neoadjuvant chemotherapy combining DCE-MRI and apparent diffusion coefficient values in breast Cancer

**DOI:** 10.1186/s12885-022-10315-x

**Published:** 2022-12-02

**Authors:** Xinhong Liang, Xiaofeng Chen, Zhiqi Yang, Yuting Liao, Mengzhu Wang, Yulin Li, Weixiong Fan, Zhuozhi Dai, Yunuo Zhang

**Affiliations:** 1 Department of Radiology, Meizhou People’s Hospital, Meizhou, 514031 China; 2 GE Healthcare, Guangzhou, 510623 China; 3 MR Scientific Marketing, Siemens Healthineers, Guangzhou, 510620 China; 4 Department of Radiology, Shantou Central Hospital, Guangdong, 515041 China; 5 Department of Oncology, Meizhou People’s Hospital, Meizhou, 514031 China

**Keywords:** Neoadjuvant chemotherapy, DCE-MRI, apparent diffusion coefficient values, treatment response, breast cancer

## Abstract

**Introduction:**

Improving the early prediction of neoadjuvant chemotherapy (NAC) efficacy in breast cancer can lead to an improved prediction of the final prognosis of patients, which would be useful for promoting individualized treatment. This study aimed to explore the value of the combination of dynamic contrast-enhanced (DCE)-MRI parameters and apparent diffusion coefficient (ADC) values in the early prediction of pathological complete response (pCR) to NAC for breast cancer.

**Methods:**

A total of 119 (range, 28−69 years) patients with biopsy-proven breast cancer who received two cycles of NAC before breast surgery were retrospectively enrolled from our hospital database. Patients were divided into pCR and non pCR groups according to their pathological responses; a total of 24 patients achieved pCR, while 95 did not. The quantitative (K^trans^; K_ep_; V_e_; IAUC) and semiquantitative parameters (W-in; W-out; TTP) of DCE-MRI that were significantly different between groups were combined with ADC values to explore their value in the early prediction of pCR to NAC for breast cancer. The independent T test was performed to compare the differences in DCE-MRI parameters and ADC values between the two groups. Receiver operating characteristic (ROC) curves were plotted, and the area under the ROC curve (AUC), sensitivity and specificity were calculated to evaluate the performance of the prediction.

**Results:**

The K^trans^, K_ep_, IAUC, ADC, W-in and TTP values were significantly different between the pCR and non pCR groups after NAC. The AUC (0.845) and specificity (95.79%) of the combined K^trans^, K_ep_, IAUC and ADC values were both higher than those of the individual parameters. The combination of W-in, TTP and ADC values had the highest AUC value (0.886) in predicting pCR, with a sensitivity and specificity of 87.5% and 82.11%, respectively.

**Conclusions:**

The results suggested that the combination of ADC values and quantitative and semiquantitative DCE-MRI parameters, especially the combination of W-in, TTP, and ADC values, may improve the early prediction of pCR in breast cancer.

## Introduction

Breast cancer is one of the most common malignancies in women, posing a threat to both their physical and mental health [1, 2]. Neoadjuvant chemotherapy (NAC) is widely used in the treatment of early and locally advanced breast cancer [3]. It can help increase the possibility of breast-conserving surgery by reducing the clinicopathological stage of breast cancer so that more patients can achieve an improved prognosis [4]. Patients who obtain a pathologic complete response (pCR) have better overall survival and disease-free survival results than those who do not [5]. A pCR can be used as a surrogate endpoint for the prediction of long-term clinical benefits of treatment [6, 7]. Improving the early prediction of NAC efficacy in breast cancer can lead to an improved prediction of the final prognosis of patients, which would be useful for promoting individualized treatment.

Currently, changes in tumor size and Ki67 are used to assess the tumor response to NAC. However, morphological changes occur later than pharmacokinetic alterations [8]. The tumor response is related to estrogen receptor status and the Ki67 proliferation index. Unfortunately, it is mainly assessed with core biopsy specimens. Moreover, samples have spatial heterogeneity and thus may not represent the entire tumor [9].

Dynamic contrast-enhanced MRI (DCE-MRI) is a noninvasive and comprehensive examination that can show changes in tumor microcirculation and angiogenesis, while changes in the ADC value can reflect cellular damage or cellular proliferation. DCE-MRI semiquantitative parameters (including W-in, W-out, TTP) and quantitative parameters (including K^trans^, K_ep_, V_e_, IAUC) can reflect blood flow, vascular density, vessel permeability, and tissue volume fractions, which were shown to be good early predictors for NAC [10–14]. Ah-See *et al*. showed that change of K^trans^ was the best predictor for PCR [13]. An increase in Ve has been shown to be a poor predictor for NAC [12]. W-in was associated with pCR rates in HER2-positive breast cancer [14]. S.Y. Hahn's research showed that the addition of DWI to DCE-MRI significantly improved the diagnostic performance in predicting the pathologic response [15].

Compared with morphological examination alone, DCE-MRI combined with ADC can be used earlier and is more accurate and objective in evaluating the efficacy of NAC for breast cancer. However, the imaging principles of DCE-MRI and DWI are different; they have their own characteristics and advantages, and applying them separately is suboptimal. By combining DCE-MRI and DWI, each can compensate for the other’s shortcomings in the evaluation of the efficacy of NAC for breast cancer. In addition, there are few studies on the simultaneous investigation of the quantitative and semiquantitative parameters of DCE-MRI [12]. Therefore, this study aims to investigate the early predictive value of the quantitative and semiquantitative parameters of DCE-MRI combined with ADC values for the pCR to NAC in breast cancer.

## Methods

### Study population

The ethics committee of Meizhou People’s Hospital approved this retrospective study (2019-C-65), and the requirement for informed consent was waived. All methods were performed in accordance with the ethical standards of the institutional and/or national research committee and with the Declaration of Helsinki [16, 17]. We retrospectively reviewed breast cancer patients with biopsy-proven breast cancer who received neoadjuvant chemotherapy before breast surgery from our hospital between July 2015 and January 2019. Patient Inclusion Criteria: ①An initial diagnosis of unilateral breast cancer and administration of neoadjuvant chemotherapy. ② MRI examination within 2 weeks before NAC (pre-NAC MRI) and after the second cycle of NAC (post-NAC MRI). ③Surgical resection of the lesion within 3 weeks after NAC and determination of the pathological results. Patient Exclusion Criteria: ①Lack of baseline or post-NAC MR data. ②Artefact that affected visualization of the lesion. ③Previous breast cancer treatment (e.g., surgery, chemotherapy or radiation therapy). To avoid treatment delay, all patients underwent MRI examination without considering the menstrual cycle. Ultimately, 119 patients fulfilled our inclusion criteria. All the patients were female (range, 28−69 years). Patients were divided into a pCR group and a non pCR group according to their pathological response. Menopausal status, baseline maximum tumor diameter, histological type, estrogen receptor (ER), progesterone receptor (PR), human epidermal growth factor receptor type 2 (HER2), Ki67%, molecular subtypes, clinical T/N stage, neoadjuvant treatment protocol, pathological response, quantitative and semiquantitative DCE-MRI parameters, and ADC values were recorded. Ki-67 ≥20% indicated high expression [18, 19]. Premenopause refers to the whole reproductive period before menopause. Postmenopause is defined as the period dating from the final menstrual period, regardless of whether menopause is induced or spontaneous [20]. Luminal A was defined as positive for ER and/or PR, HER-2-negative, and Ki-67<20%. Luminal B was defined as ER- and/or PR-positive, HER-2-positive or-negative, while Ki-67 was ≥20%. HER-2 enrichment was defined as ER-and PR-negative with HER-2 positivity. TNBC was defined as all negative for ER, PR, and HER- 2[2].

### Imaging Protocol

All breast MR examinations were performed with a 3.0T MRI scanner (Siemens, Germany) in the prone position with the bilateral breasts naturally sagging within a 16-channel phased-array breast coil. DCE-MRI was performed using a 3D combination of volume interpolated breath-hold (VIBE) with controlled aliasing in parallel imaging results in higher acceleration (CAIPIRINHA), including view-sharing time-resolved imaging with interleaved stochastic trajectories (TWIST) and Dixon fat suppression (CAIPIRINHA-Dixon-TWIST-VIBE), a spoiled gradient echo sequence with a fast low angle shot, and the K-space sharing technology TWIST. The DCE sequence consisted of precontrast T1-weighted VIBE imaging (repetition time (TR)=3.78 ms, echo time (TE)=1.38 ms, voxel resolution=1.3 mm×1.3 mm×2.0 mm, matrix=205×256, slice thickness=2 mm, field of view (FOV)=340 mm×340 mm) and TWIST-VIBE DCE scanning with 34 consecutive phases (TR=6.4 ms, TE=3.34 ms, voxel resolution=0.9 mm×0.9 mm×2.0 mm, matrix=288×384, FOV=340 mm×340 mm, slice thickness=2 mm, no slice gap, flip angle=9°, temporal resolution= 8.7 s). Gadopentetate dimeglumine (Bayer Pharma AG) was intravenously injected at a dose of 0.1 mmol/kg with an injection flow rate of 3.0 ml/s after phase 1 and 2 mask scanning. Then, 20 ml normal saline was injected at the same flow rate. DWI images were sequentially obtained by readout-segmented (RS) echo-planar imaging (EPI) and single-shot (SS)-EPI techniques with fat suppression in the transverse plane before DCE-MRI. The parameters of RS-EPI were as follows: TR/TE 4800/56 ms, FOV 170 mm×340 mm, matrix 96×192, FA 180°, bandwidth 868 Hz, slice thickness 4.0 mm, slice gap 0.8 mm, number of averages 8, and two b-values (50 and 800 s/mm^2^). The parameters of SS-EPI were as follows: TR/TE 4200/62 ms, FOV 149 mm×340 mm, matrix 100×170, bandwidth 1730 Hz, echo spacing 0.68 ms, slice thickness 4.0 mm, slice gap 0.8 mm, number of averages 3, and two b-values (50 and 800 s/mm^2^). In addition, generalized auto calibrating, partially parallel acquisitions were used in both sequences with an acceleration factor of 2, and sufficient slices were acquired to cover the entire breast.

### Data processing and collection

**Fig. 1:**
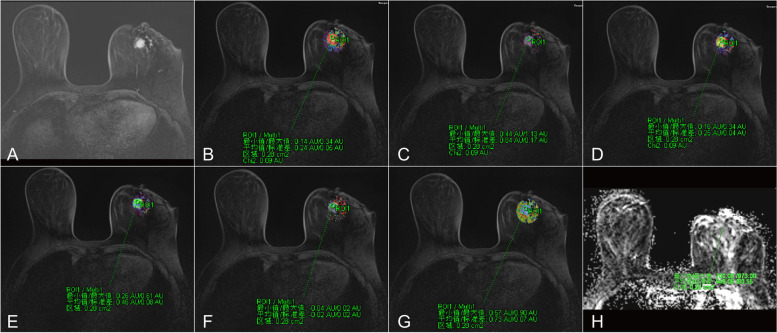
MR images of a 65-year-old woman with invasive ductal carcinoma of the left breast before NAC. **A** TWIST-DCE image. **B**–**H** Pseudocolor images for determining K^trans^ (**B**), K_ep_ (**C**), IAUC (**D**), W-in (**E**), W-out (**F**), TTP (**G**), and ADC (**H**). Red represents high values, yellow intermediate values, and blue low values. The values for K^trans^, K_ep_, IAUC, W-in, W-out, and TTP were 0.309 min^-1^, 1.136 min^-1^, 0.336, 0.499 min^-1^, 0.013 min^-1^, 0.582 minutes and 0.849×10^-3^ mm^2^/s, respectively

**Fig. 2:**
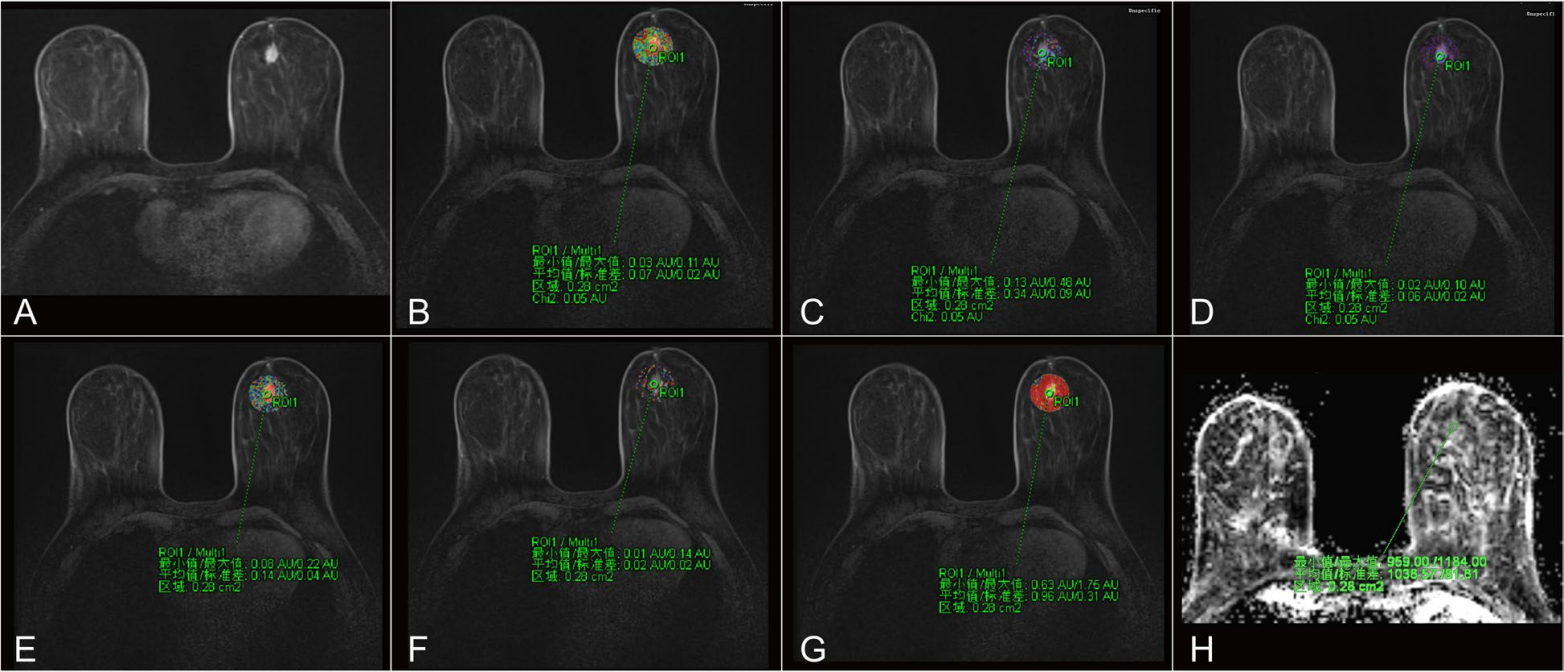
MR images of the same patient after two cycles of NAC. The patient underwent surgery after six cycles of NAC. Surgical specimens indicated a complete response (Miller-Payne grade 5). **A** TWIST-DCE image. **B**–**H** Pseudocolor images for K^trans^ (**B**), K_ep_ (**C**), IAUC (**D**), W-in (**E**), W-out (**F**), TTP (**G**), and ADC (**H**). Red represents high values, yellow intermediate values, and blue low values. The values for K^trans^, K_ep_, IAUC, W-in, W-out, and TTP were 0.069 min^-1^, 0.339 min^-1^, 0.083, 0.181 min^-1^, 0.022 min^-1^, 0.868 minutes and 1.036×10^-3^ mm ^2^/s, respectively

All MRI data were independently analyzed by 2 radiologists with more than ten years of breast MRI experience who were blinded to the patient's characteristics. The patient images were imported into a Siemens supporting workstation. DCE-derived parametric maps of quantitative and semiquantitative parameters were automatically generated after motion correction on the Tofts model and qualitative model, respectively, using the MR Tissue 4D software tool (Siemens Healthcare). The arterial input function (AIF) was obtained by population averaging 50 individual AIFs obtained from breast cancer patients scanned at different time points. The lesion location was determined by combining the T2W, DW and DCE-MRI images. Each parameter was measured 3 times with a region of interest (ROI) of a minimum area of 10 mm^2^, placed on the largest tumor section and its adjacent sections, and the averaged value was then calculated for further analysis. Tumors displaying a signal intensity increase of greater than 80% were defined as the voxels in each ROI (Figure 1 and Figure 2), as this was the optimal threshold enhancement level determined by a previous study [21]. The threshold was calculated as ((Spost−Spre)/Spre) × 100. The ROI was first drawn on the volume transfer constant (K^trans^) derivative map and automatically generated onto the other quantitative parameter derivative maps. The ROI of each parameter derivative map encompassed the same position and range. Then, the same ROI was matched to the semiquantitative parameter derivative and ADC maps while ensuring that the ADC values of the DWI (b= 800 s/mm^2^) had the same ROI as the DCE-derived parametric maps. Care was taken to avoid including vascular structures, calcifications, hemorrhage, cystic areas, necrotic areas, normal breast parenchyma and fat. All ROIs were drawn on DCE-derived parametric maps and ADC maps by two experienced breast radiologists. The mean values of ADC, K^tran^, K_ep_, V_e_, IAUC, W-in, W-out, and TTP were used for further analysis. The quantitative parameters of DCE-MRI were as follows: (1) K^trans^; (2) K_ep_; (3) V_e_; and (4) Initial area under the curve (IAUC). K^trans^ refers to the rate at which the contrast agent diffuses from the intravascular to the extravascular space (unit, min^-1^). K_ep_ refers to the rate at which the contrast agent flows back into the blood vessel from the extracellular space (unit, min^-1^). V_e_ refers to the extravascular space per unit volume of tissue and is calculated as V_e_= K^trans^/ K_ep_ (unit, %). IAUC refers to the initial area under the curve for the first 60 seconds. The semiquantitative parameters of DCE-MRI were as follows: (1) W-in; (2) W-out; and (3) TTP. W-in refers to the rate of contrast enhancement for contrast agent inflow (unit, min^-1^). W-out refers to the rate of contrast decay for contrast agent outflow (unit, min^-1^). TTP refers to the time-to-peak enhancement after contrast agent injection (units, min).

### Pathological Assessment

The Miller-Payne grading system [22] is a 5-level classification method used to evaluate the pathological response. Grade 5: no identifiable malignant cells seen in the sections, although ductal carcinoma in situ may exist. Grades 1 to 4 were categorized as a non pCR, and grade 5 was categorized as pCR.

### Statistical analysis

Statistical analysis was performed with MedCalc software (MedCalc Inc., Mariakeke, Belgium). The chi-square test was used to compare the differences in clinical characteristics between the pCR and non pCR groups. The Kolmogorov–Smirnov test was used to assess the normality of the parameters. For parameters that were normally distributed, the independent-samples T test was used for comparisons between groups. The Mann–Whitney U test was used for parameters with a nonnormal distribution. The DCE-MRI quantitative parameters (K^trans^; K_ep_; V_e_; IAUC) and semiquantitative parameters (W-in; W-out; TTP) that were significantly different between groups were combined with ADC values. Using postoperative histopathological diagnosis as the gold standard, the receiver operating characteristic (ROC) curve was used to predict the pCR of breast cancer after NAC. The Youden index was used for threshold division in this study. The AUC, sensitivity, specificity, and positive- and negative-predictive values of each parameter were calculated. Correlations between quantitative parameters and semiquantitative parameters were analyzed using Spearman's test. Differences were considered statistically significant when the *P* value was less than 0.05.

## Results

### Clinical Characteristics

All the patients were female with an age range of 28−69 years. Among the 119 patients, the average age was 49.3 years ± 9.8 (standard deviation). The patient and tumor characteristics are presented in Table 1. In this study, the patients were divided into two groups according to the Miller & Payne system; the pCR rate was 20.2%. There was a significant difference in ER, PR, Ki67 status, and molecular subtypes and no significant differences in age, baseline maximum tumor diameter, HER-2 status, clinical T/N stage, menopausal status, histological type, or neoadjuvant treatment protocol between the two groups.

**Table 1:** Baseline Characteristics of the Study Population

Characteristics	pCR (*n=*24)	Non pCR (*n=*95)	*P* Value
Age (y)			
≤50	12	48	0.963
>50	12	47	
Baseline maximum Tumor diameter (mm)			
<20	0	2	0.592
20-50	16	51	
>50	8	42	
Clinical T stage			
cT1	0	4	0.261
cT2	14	38	
cT3	5	36	
cT4	5	17	
Clinical N stage			
cN0	0	0	0.062
cN1	4	40	
cN2	10	26	
cN3	10	29	
Receptor status			
ER(+)	7	67	<0.001*
ER(-)	17	28	
PR(+)	2	44	<0.001*
PR(-)	22	51	
HER-2			
(+)	11	40	0.742
(-)	13	55	
Ki67%			
≤20%	2	45	<0.001*
>20%	22	50	
molecular subtypes			
Luminal A	0	11	<0.001*
Luminal B1	1	37	
Luminal B2	5	18	
HER2	6	22	
triple negative	12	7	
Menopausal status			
Postmenopausal	12	42	0.611
Premenopausal	12	53	
Histological type			
Invasive ductal carcinoma	24	91	0.582
Other	0	4	
Neoadjuvant treatment protocol			
TC	1	1	0.197
FEC-T	1	1	
TEC	18	82	
EC-T	3	10	
Other	1	1	

Note:*P value <0.05, indicating that the differences were considered statistically significant. Abbreviations: *ER* Estrogen receptor, PR Progesterone receptor, HER2 Human epidermal growth factor receptor type 2, *T* Docetaxel, *C* Cyclophosphamide, *F* 5-Fluorouracil, *E* Epirubicin

### Comparison of MRI parameters before and after NAC

Analysis of the imaging parameters revealed that the pre-NAC V_e_, ADC and W-out conformed to a normal distribution, while the other parameters were nonnormally distributed both pre- and post-NAC. None of the pre-NAC parameters were significantly different (all *P* > 0.05) between the pCR and non pCR groups (Table 2).

**Table 2:** Comparison of pre-NAC DCE-MRI parameters and ADC values between pCR and non pCR group

Groups	N	K^trans†^ (min^-1^)	K_ep_^†^ (min^-1^)	V_e_^#^ (%)	IAUC^†^	ADC^#^	W-in^†^	W-out^#^	TTP^†^
Non pCR	95	0.18 (0.14, 0.24)	0.69 (0.53, 0.85)	0.28±0.09	0.21 (0.15, 0.26)	0.79±0.16	0.42 (0.28, 0.53)	-0.004±0.02	0.58 (0.58, 0.72)
pCR	24	0.18 (0.12, 0.25)	0.67 (0.49, 0.92)	0.29±0.10	0.25 (0.14, 0.28)	0.83±0.15	0.42 (0.28, 0.56)	0.001±0.02	0.58 (0.58, 0.72)
P Value		0.761^b^	0.866^b^	0.82^a^	0.674^b^	0.22^a^	0.992^b^	0.26^a^	0.458^b^

Note:^#^Results are the mean value with standard deviation. ^†^Results are the median with interquartile range in parentheses. *P*^a^: independent-samples T test. *P*^b^: Mann–Whitney U test. Abbreviations: *N* Number, *pCR* Pathologic complete response

**Fig. 3:**
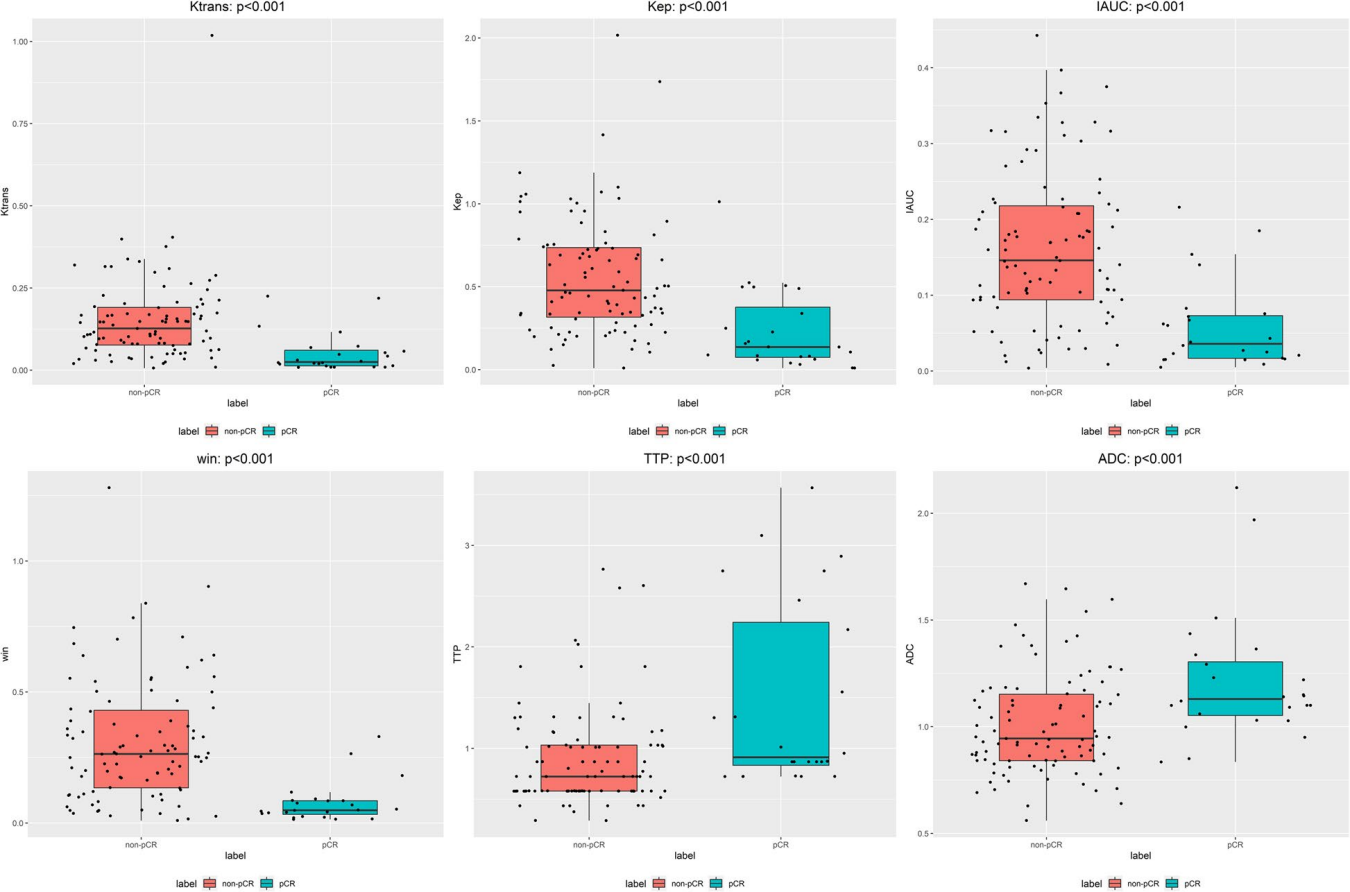
Box plot graphs revealing statistically significant differences in values between CR and non pCR groups

The ADC values increased significantly, and the values of K^trans^, K_ep_, and IAUC decreased significantly in the pCR group after NAC (Table 3, Fig. 3). W-in and TTP were significantly different between the pCR and non pCR groups after NAC. In the pCR group, W-in decreased and TTP increased after NAC. However, V_e_ and W-out were not significantly different between the pCR and non pCR groups after NAC.

**Table 3:** Comparison of post-NAC DCE-MRI parameters and ADC values between pCR and the non pCR group

Groups	N	K^trans†^ (min^-1^)	K_ep_^†^ (min^-1^)	V_e_^†^ (%)	IAUC^†^	ADC^†^	W-in^†^	W-out^†^	TTP^†^
Non pCR	95	0.13 (0.08, 0.19)	0.48 (0.31, 0.74)	0.24 (0.19, 0.36)	0.15 (0.09, 0.22)	0.94 (0.84, 1.15)	0.26 (0.13, 0.43)	0.00(-0.01, 0.02)	0.72 (0.58, 1.03)
pCR	24	0.03 (0.01, 0.06)	0.14 (0.07, 0.42)	0.23 (0.15, 0.39)	0.04 (0.02, 0.07)	1.13 (1.04, 1.32)	0.05 (0.03, 0.09)	0.01(-0.00, 0.02)	0.91 (0.79, 2.33)
P Value		<0.001^b^*	<0.001^b^*	0.527^b^	<0.001^b^*	0.001^b^*	<0.001^b^*	0.276^b^	0.001^b^*

Note:^†^Results are the median with interquartile range in parentheses. P^b^: Mann–Whitney U test. *P value <0.05, indicating that the differences were considered statistically significant. Abbreviations: *N* Number, *pCR* Pathologic complete response

### Diagnostic performance of individual models in predicting pCR

ROC curve analysis was conducted for the quantitative and semiquantitative DCE-MRI parameters and ADC values before and after two cycles of NAC in predicting pCR. As seen in Table 4, those parameters before NAC could not predict the diagnostic efficacy of NAC.

**Table 4:** Diagnostic performance of parameters in predicting pCR before NAC

Parameter	AUC	Cutoff	Sensitivity	Specificity	*P* Value
K^trans^ (min^-1^)	0.52	≤0.139	37.50	74.74	0.771
K_ep_ (min^-1^)	0.511	≤0.548	41.67	72.63	0.881
V_e_(%)	0.562	>0.291	62.50	63.16	0.365
IAUC	0.528	>0.244	54.17	66.32	0.689
ADC	0.607	>0.82	66.67	63.16	0.097
W-in	0.501	≤0.326	41.67	71.58	0.993
W-out	0.549	>0.014	33.33	85.26	0.512
TTP	0.549	≤0.582	70.83	41.05	0.440

**Fig. 4:**
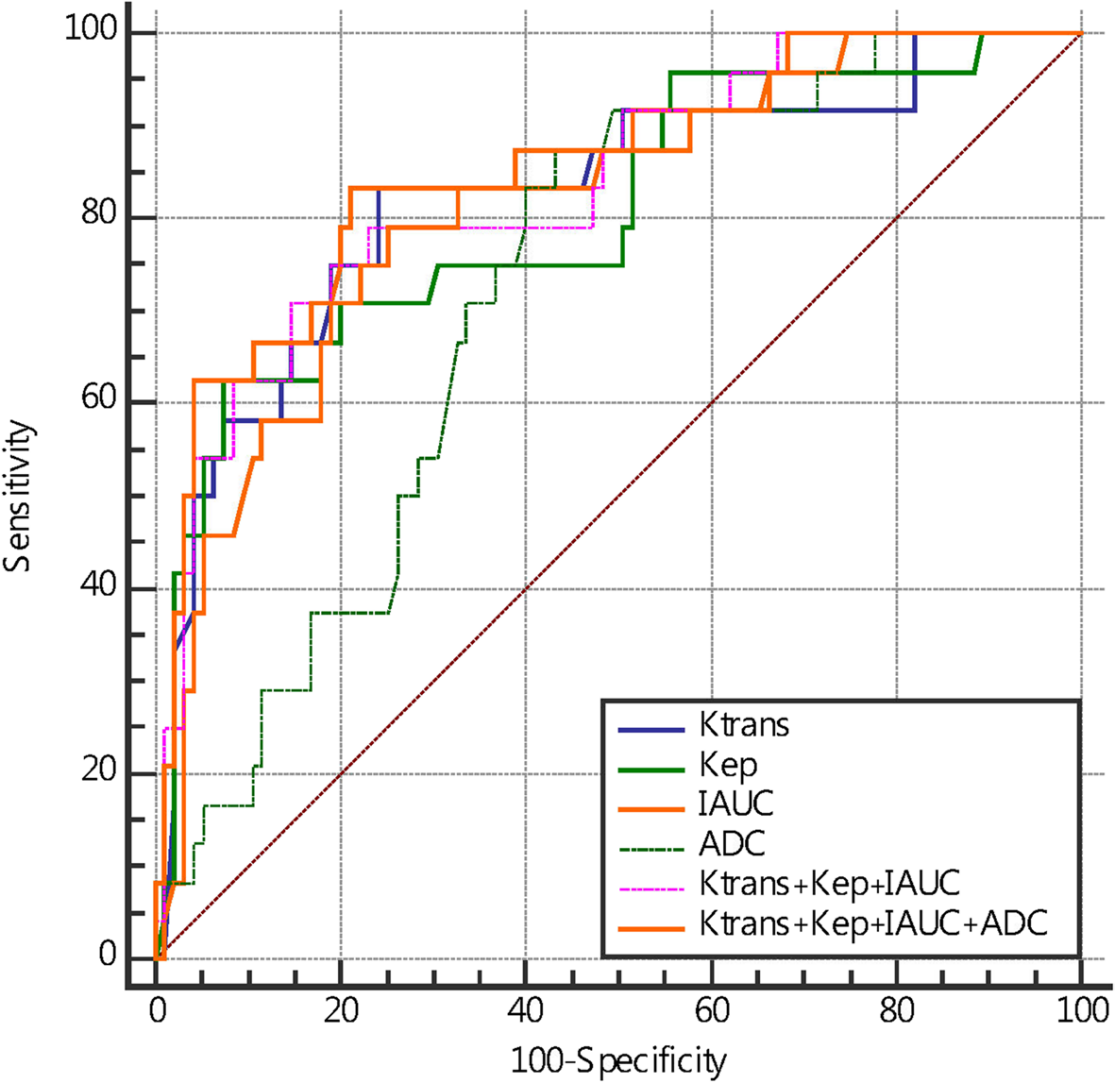
ROC curves of DCE-MRI quantitative parameters, ADC value, and combination of multiple parameters in predicting pCR after two cycles of NAC

However, K^trans^, K_ep_, IAUC, ADC values, W-in and TTP post-NAC showed fair to good predictive performances for pCR (Table 5). W-in had the highest diagnostic efficiency, K_ep_ had the highest specificity, and TTP had the highest sensitivity; however, the specificity of TTP was relatively low. The differences in V_e_ and W-out between the pCR and non pCR groups were not significant. The specificities of K^trans^, IAUC and W-in were all relatively high. The AUC of the combination of quantitative DCE-MRI parameters K^trans^, K_ep_ and IAUC in predicting pCR after NAC for breast cancer was greater than individual performance (Figure 4).

**Table 5:** Diagnostic performance of all parameters in predicting pCR after NAC

Parameters	AUC	Cutoff	Sensitivity	Specificity	PPV	NPV	*P* value
K^trans^ (min^-1^)	0.825	≤0.073	83.33	75.79	0.433	0.998	<0.001*
K_ep_ (min^-1^)	0.805	≤0.169	62.50	92.63	0.257	0.965	<0.001*
V_e_(%)	0.542	≤0.204	45.83	71.58	1.064	0.919	0.572
IAUC	0.824	≤0.083	83.33	78.95	0.388	0.998	<0.001*
ADC	0.721	>0.981	87.50	56.84	0.312	0.998	<0.001*
W-in	0.866	≤0.092	83.33	84.21	1.095	0.397	<0.001*
W-out	0.572	>0.009	62.50	58.95	0.623	0.405	0.238
TTP	0.725	>0.582	100.00	42.11	0.304	0.457	<0.001*
K^trans^ + K_ep_ +IAUC	0.836	>-0.721	70.83	85.26	0.642	0.724	<0.001*
K^trans^+K_ep_+IAUC+ADC	0.845	>-0.223	62.50	95.79	0.657	0.973	<0.001*
W-in+TTP	0.865	>-0.684	83.33	84.21	0.486	0.735	<0.001*
W-in+TTP+ADC	0.886	>-0.811	87.50	82.11	0.441	0.711	<0.001*

*P value <0.05, indicating that the differences were considered statistically significant. Abbreviations: *PPV* Positive predictive value, *NPV* Negative predictive value

**Fig. 5:**
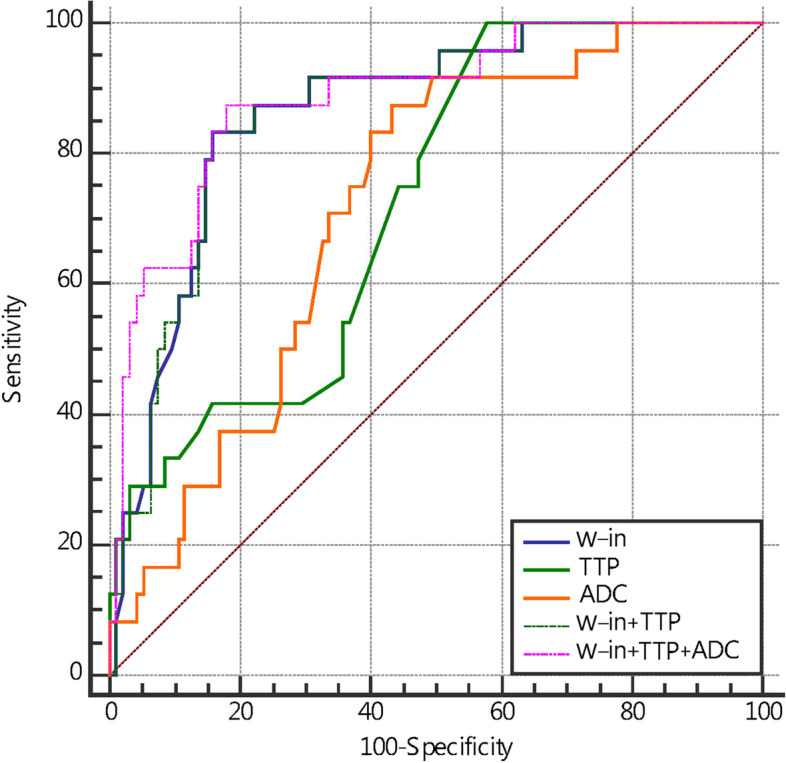
ROC curves of DCE-MRI semiquantitative parameters, ADC values and combinations of multiple parameters in predicting pCR after NAC

**Fig. 6:**
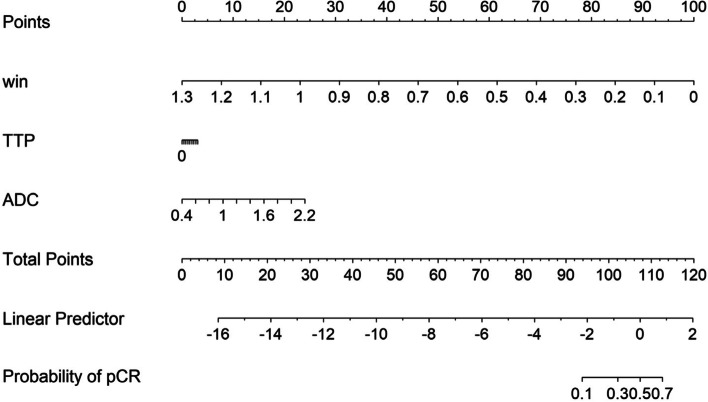
The nomogram to predict pCR with quantitative DCE- MRI parameters and ADC values after two cycles of NAC for breast cancer patients

The AUC and specificity of the combined K^trans^, K_ep_, IAUC, and ADC values in predicting pCR after NAC in breast cancer were further increased, but the sensitivity decreased. The AUC of the combined semiquantitative DCE-MRI parameters W-in and TTP in predicting pCR after NAC for breast cancer increased (Figure 5) and was higher than that of TTP alone (Delong test *P*=0.004). The combination of W-in, TTP and ADC value had the highest AUC, and the corresponding nomogram was developed (Figure 6).

## Discussion

In this study, we compared and analyzed the application of ADC values and quantitative and semiquantitative DCE-MRI parameters alone and in various combinations in predicting the efficacy of NAC in breast cancer. Our study showed that there were no significant differences in the quantitative or semiquantitative DCE-MRI parameters or ADC values before NAC between the pCR group and non pCR group. The ADC values and TTP increased significantly, and the values of K^trans^, K_ep_, IAUC and W-in decreased significantly in the pCR group after two cycles of NAC. In the pCR group, tumor cell necrosis and the decrease in the number of tumor vessels were more obvious, and the microvascular perfusion was lower, resulting in a more significant decrease in K^trans^, K_ep_, IAUC and W-in values and a longer TTP than in the non pCR group after NAC. In addition, water molecule movement was enhanced, so the increase in the ADC value was more obvious.

A recent study from Zhao *et al* showed that the combination of DCE-MRI and DWI could successfully predict pCR after two cycles of NAC, in which changes in DCE-MRI and DWI parameters before and after NAC were used [23]. In contrast to a prior study by Zhao *et al*, we evaluated the DCE-MRI and DWI parameters before and after NAC, and our study showed that none of the pre-NAC parameters were significantly different between the pCR and non pCR groups, the ADC values increased significantly, and the values of K^trans^, Kep, and IAUC decreased significantly in the pCR group after NAC. In addition, K^trans^, K_ep_, IAUC, ADC values, W-in and TTP post-NAC showed fair to good predictiveness for pCR. Specifically, patients with ADC > 0.981×10^-3^ mm^2^/s, TPP > 0.582 min, and K^trans^ ≤ 0.073 min^-1^ were more likely to reach pCR. Furthermore, our results showed that V_e_ and W-out did not differ significantly between the two groups after NAC, suggesting that they are poor indicators of the response. M. Pickles [24] considered the V_e_ value to be affected by edema around the lesion, resulting in unstable measurements. This could explain the nonsignificant difference in V_e_ between the two groups. Our results showed that DWI and DCE-MRI reflect the changes in the internal components of the lesion, and MRI monitoring of the response during NAC was effective for breast cancer, consistent with previous studies [15, 21, 25].

The more significant the decrease in neovascularization and vascular permeability is, the lower the microvascular perfusion, which may be the reason why the K^trans^ and K_ep_ decreased more significantly in the pCR group than in the non pCR group. A. Saracco's [26] research showed that the TTP was significantly longer in those who were treated effectively than in those who were not treated after two cycles of NAC, which was similar to our results. They considered this to mean that decreased blood perfusion will slow the blood flow in the tumor. Additionally, we also found that the sensitivity of TTP was 1. The Youden index was used for threshold division in this study. Depending on clinical practice, sensitivity and specificity may vary if other methods are used. The main reason for the high sensitivity was that the number of pCR patients was smaller than the number of non pCR patients in our study. The increase in ADC values after NAC was the result of cellular damage and necrosis. During the apoptotic process, cell lysis and cell membrane blebbing result in initial cellular swelling, followed by a reduction in cellular volume. Then, the movement of water molecules is enhanced, and water can move more freely between the intra- and extracellular space [25]. This may account for the higher ADC values in the pCR group than in the non pCR group. Therefore, it has more advantages in the efficacy evaluation of patients with no obvious changes in tumor volume and is more accurate and objective in the efficacy evaluation of breast cancer after NAC. The microscopic changes mentioned above were more obvious in the pCR group than in the non pCR group. The AUC value of the combined K^trans^, K_ep_, IAUC and ADC values in predicting pCR after NAC in breast cancer further increased to 0.845, and the specificity increased to 95.79%. The AUC and specificity of the combination of K^trans^, K_ep_, IAUC and ADC values were both higher than those of the individual diagnoses, indicating that this combination is desirable. The combination of W-in, TTP and ADC values post-NAC for breast cancer had the highest AUC value in predicting pCR, with a sensitivity and specificity of 87.5% and 82.11%, respectively, which improved the imbalance of sensitivity and specificity when using the single parameters.

Overall, the combination of DCE-MRI parameters and ADC values may be a good early predictor of pathologic response, and its diagnostic efficiency was higher than that of either the DCE-MRI parameters or ADC values alone. Multiparametric MRI may thus better reflect information on the tumor. S.Y. Hahn's [15] research showed that the addition of DWI to DCE-MRI significantly improved the diagnostic performance in predicting pathologic response. This is similar to our findings. X. Li's [21] research showed that the multiparametric analysis of DCE-MRI and DWI was superior to the single-parameter measurements in predicting pCR after the first cycle of NAC. This is also similar to the results of our study, except that our study compared the parameters after two cycles of NAC. The reason may be that the combined diagnosis combined the imaging advantages of DCE-MRI and ADC. Therefore, to provide more accurate and comprehensive diagnostic information for breast cancer patients undergoing neoadjuvant chemotherapy, DCE-MRI and ADC should be combined for preoperative evaluation.

There are several limitations to our study. First, to avoid treatment delay, all patients underwent MRI examination without considering the menstrual cycle. Second, because this was a retrospective study and the sample size was small, we could not control for possible selection bias. Third, our results may be affected by the contrast agent and scanning protocol. In this study, the same scanning sequence and the same contrast agent were used. In the future, we will expand the research with different contrast agents and scanning protocols. Additionally, the treatments were heterogeneous, with chemotherapy schemes varying. This could be an unpredictable factor affecting the results. In the future, prospective studies with a larger sample size are warranted.

## Conclusions

In conclusion, ADC values combined with quantitative and semiquantitative DCE-MRI parameters may improve the early prediction of pCR to NAC in breast cancer, particularly the combination of W-in, TTP and ADC values. The semiquantitative parameters (W-in, TTP) were obtained directly from the description of the signal intensity time curve, which is easily obtained. Noninvasive and individualized preoperative prediction of pCR after NAC can help promote personalized treatment. Treatment strategies and surveillance monitoring may be better guided, and this may result in a higher rate of complete response at the end of treatment.

## Data Availability

The datasets used and/or analyzed during the current study are available from the corresponding author on reasonable request.

## Abbreviations

DCE-MRI: Dynamic contrast-enhanced MRI
ADC: Apparent diffusion coefficient
pCR: Pathological complete response
ROC: Receiver operating characteristic
AUC: Area under the ROC curve
NAC: Neoadjuvant chemotherapy
DWI: Diffusion-weighted imaging
ER: Estrogen receptor
PR: Progesterone receptor
HER2: Human epidermal growth factor receptor type 2

## References

[1] Ryu JM, Lee SK, Kim JY, Yu J, Kim SW, Lee JE, Han SH, Jung YS, Nam SJ (2017). Predictive Factors for Nonsentinel Lymph Node Metastasis in Patients With Positive Sentinel Lymph Nodes After Neoadjuvant Chemotherapy: Nomogram for Predicting Nonsentinel Lymph Node Metastasis. Clinical breast cancer.

[2] Yang Z, Chen X, Zhang T, Cheng F, Liao Y, Chen X, Dai Z, Fan W (2021). Quantitative Multiparametric MRI as an Imaging Biomarker for the Prediction of Breast Cancer Receptor Status and Molecular Subtypes. Frontiers in oncology.

[3] Chen X, Chen X, Yang J, Li Y, Fan W, Yang Z (2020). Combining Dynamic Contrast-Enhanced Magnetic Resonance Imaging and Apparent Diffusion Coefficient Maps for a Radiomics Nomogram to Predict Pathological Complete Response to Neoadjuvant Chemotherapy in Breast Cancer Patients. J Comput Assist Tomogr.

[4] Fowler AM, Mankoff DA, Joe BN (2017). Imaging Neoadjuvant Therapy Response in Breast Cancer. Radiology.

[5] Dong JM, Wang HX, Zhong XF, Xu K, Bian J, Feng Y, Chen L, Zhang L, Wang X, Ma DJ (2018). Changes in background parenchymal enhancement in HER2-positive breast cancer before and after neoadjuvant chemotherapy: Association with pathologic complete response. Medicine (Baltimore).

[6] Pennisi A, Kieber-Emmons T, Makhoul I, Hutchins L (2016). Relevance of Pathological Complete Response after Neoadjuvant Therapy for Breast Cancer. Breast cancer : basic and clinical research.

[7] Cho HH, Park M, Park H, Ko ES, Hwang NY, Im YH, et al. The Tumor-Fat Interface Volume of Breast Cancer on Pretreatment MRI Is Associated with a Pathologic Response to Neoadjuvant Chemotherapy. Biology. 2020;9(11). 10.3390/biology9110391 PMC7697338 33182628

[8] Pereira NP, Curi C, Osório C, Marques EF, Makdissi FB, Pinker K, Bitencourt AGV (2019). Diffusion-Weighted Magnetic Resonance Imaging of Patients with Breast Cancer Following Neoadjuvant Chemotherapy Provides Early Prediction of Pathological Response - A Prospective Study. Scientific reports.

[9] Yip SS, Aerts HJ (2016). Applications and limitations of radiomics. Physics in medicine and biology.

[10] Tudorica A, Oh KY, Chui SY, Roy N, Troxell ML, Naik A, Kemmer KA, Chen Y, Holtorf ML, Afzal A (2016). Early Prediction and Evaluation of Breast Cancer Response to Neoadjuvant Chemotherapy Using Quantitative DCE-MRI. Translational oncology.

[11] Li X, Arlinghaus LR, Ayers GD, Chakravarthy AB, Abramson RG, Abramson VG, Atuegwu N, Farley J, Mayer IA, Kelley MC (2014). DCE-MRI analysis methods for predicting the response of breast cancer to neoadjuvant chemotherapy: pilot study findings. Magnetic resonance in medicine.

[12] Sharma A, Sharma S, Sood S, Seam RK, Sharma M, Fotedar V (2018). DCE-MRI and parametric imaging in monitoring response to neoadjuvant chemotherapy in breast carcinoma: a preliminary report. Polish J Radiol.

[13] Ah-See ML, Makris A, Taylor NJ, Harrison M, Richman PI, Burcombe RJ, Stirling JJ, d'Arcy JA, Collins DJ, Pittam MR (2008). Early changes in functional dynamic magnetic resonance imaging predict for pathologic response to neoadjuvant chemotherapy in primary breast cancer. Clinical cancer research : an official journal of the American Association for Cancer Research.

[14] Ramtohul T, Tescher C, Vaflard P, Cyrta J, Girard N, Malhaire C, et al. Prospective Evaluation of Ultrafast Breast MRI for Predicting Pathologic Response after Neoadjuvant Therapies. Radiology. 2022:220389. 10.1148/radiol.220389 35880977

[15] Hahn SY, Ko EY, Han BK, Shin JH, Ko ES (2014). Role of diffusion-weighted imaging as an adjunct to contrast-enhanced breast MRI in evaluating residual breast cancer following neoadjuvant chemotherapy. Eur J Radiol.

[16] Chen X, Yang Z, Yang J, Liao Y, Pang P, Fan W, Chen X (2020). Radiomics analysis of contrast-enhanced CT predicts lymphovascular invasion and disease outcome in gastric cancer: a preliminary study. Cancer imaging : the official publication of the International Cancer Imaging Society.

[17] Liu F, Wang M, Li H. Role of perfusion parameters on DCE-MRI and ADC values on DWMRI for invasive ductal carcinoma at 3.0 Tesla. World J Surg Oncol. 2018;16(1):239. 10.1186/s12957-018-1538-8 PMC6303963 30577820

[18] Guidelines for clinical diagnosis and treatment of advanced breast cancer in China (2020 Edition). Zhonghua zhong liu za zhi [Chinese journal of oncology]. 2020;42(10):781–97. 10.3760/cma.j.cn112152-20200817-00747 33113619

[19] Zhong M, Yang Z, Chen X, Huang R, Wang M, Fan W, Dai Z, Chen X (2022). Readout-Segmented Echo-Planar Diffusion-Weighted MR Imaging Improves the Differentiation of Breast Cancer Receptor Statuses Compared With Conventional Diffusion-Weighted Imaging. J Magnet Resonance Imaging : JMRI.

[20] Sherman S (2005). Defining the menopausal transition. Am J Med.

[21] Li X, Abramson RG, Arlinghaus LR, Kang H, Chakravarthy AB, Abramson VG, Farley J, Mayer IA, Kelley MC, Meszoely IM (2015). Multiparametric magnetic resonance imaging for predicting pathological response after the first cycle of neoadjuvant chemotherapy in breast cancer. Invest Radiol.

[22] Yang C, Zhao H (2020). Application of dynamic magnetic resonance imaging information technology in adjuvant chemotherapy for breast cancer. J Infect Public Health.

[23] Zhao R, Lu H, Li YB, Shao ZZ, Ma WJ, Liu PF (2022). Nomogram for Early Prediction of Pathological Complete Response to Neoadjuvant Chemotherapy in Breast Cancer Using Dynamic Contrast-enhanced and Diffusion-weighted MRI. Acad Radiol.

[24] Pickles MD, Lowry M, Manton DJ, Turnbull LW (2015). Prognostic value of DCE-MRI in breast cancer patients undergoing neoadjuvant chemotherapy: a comparison with traditional survival indicators. Eur Radiol.

[25] Xu HD, Zhang YQ (2017). Evaluation of the efficacy of neoadjuvant chemotherapy for breast cancer using diffusion-weighted imaging and dynamic contrast-enhanced magnetic resonance imaging. Neoplasma.

[26] Saracco A, Szabó BK, Tánczos E, Bergh J, Hatschek T (2017). Contrast-enhanced ultrasound (CEUS) in assessing early response among patients with invasive breast cancer undergoing neoadjuvant chemotherapy. Acta radiologica.

